# Uric Acid Produces an Inflammatory Response through Activation of NF-κB in the Hypothalamus: Implications for the Pathogenesis of Metabolic Disorders

**DOI:** 10.1038/srep12144

**Published:** 2015-07-16

**Authors:** Wenjie Lu, Youzhi Xu, Xiaoni Shao, Fabao Gao, Yan Li, Jing Hu, Zeping Zuo, Xue Shao, Liangxue Zhou, Yinglan Zhao, Xiaobo Cen

**Affiliations:** 1 State Key Laboratory of Biotherapy and Cancer Center, West China Hospital, Sichuan University, and Collaborative Innovation Center for Biotherapy, Chengdu 610041, China; 2 Department of Pathophysiology, School of Basic Medicine, Anhui Medical University, Hefei 230032, China; 3 Molecular Imaging Laboratory, Department of Radiology, West China Hospital, Sichuan University, Chengdu 610041, China; 4 National Chengdu Center for Safety Evaluation of Drugs, State Key Laboratory of Biotherapy and Cancer Center, West China Hospital, Sichuan University, and Collaborative Innovation Center for Biotherapy, Chengdu 610041, China; 5 Department of Neurosurgery, West China Hospital, Sichuan University, Chengdu 610041, China

## Abstract

Epidemiological studies have shown that an elevated uric acid (UA) level predicts the development of metabolic syndrome and diabetes; however, there is no direct evidence of this, and the underlying mechanism remains unclear. Here, we showed that a high-UA diet triggered the expression of pro-inflammatory cytokines, activated the NF-κB pathway, and increased gliosis in the hypothalamus. Intracerebroventricular injection of UA induced hypothalamic inflammation and reactive gliosis, whereas these effects were markedly ameliorated by the inhibition of NF-κB. Moreover, magnetic resonance imaging confirmed that hyperuricemia in rodents and humans was associated with gliosis in the mediobasal hypothalamus. Importantly, the rats administered UA exhibited dyslipidemia and glucose intolerance, which were probably mediated by hypothalamic inflammation and hypothalamic neuroendocrine alterations. These results suggest that UA can cause hypothalamic inflammation via NF-κB signaling. Our findings provide a potential therapeutic strategy for UA-induced metabolic disorders.

Hyperuricemia has emerged as a major health problem in industrialized nations. Epidemiological evidence has indicated that the prevalence of hyperuricemia is increasing worldwide^1^. It is estimated that 5–10% of adult Americans suffer from hyperuricemia^2^. In Asia, the prevalence of hyperuricemia is 26.1% for men and 17.0% for women^3^. Hyperuricemia has been thought to be part of the cluster of metabolic abnormalities that includes glucose intolerance and dyslipidemia^4^. Recently, elevated serum uric acid (UA) has been considered to be an independent predictor of obesity^5^, fatty liver, and diabetes^6^. Laboratory studies have shown a contributory role of UA to metabolic syndrome, which features dyslipidemia, insulin resistance, and hypertension^7^. Fructose-induced hyperuricemia induces insulin resistance, and lowering UA using xanthine oxidase inhibitors ameliorates the elevation of serum triglycerides and insulin resistance^8^. Additionally, xanthine oxidoreductase-knockout mice fail to become fat due to a defect in adipogenesis^9^. Despite this progress, little is known regarding the association of hyperuricemia with metabolic disorders and the underlying mechanism.

Growing evidence has suggested that immune-cell-mediated tissue inflammation acts as a key mechanism of metabolic disorders in metabolically active organs^10^. The hypothalamus is the “headquarters” for the regulation of energy homeostasis^11^, and hypothalamic inflammation is an early event in the development of metabolic abnormalities in peripheral tissue^12^. Hypothalamic inflammation increases baseline plasma insulin, which is accompanied by impaired insulin signaling in liver and skeletal muscle^13^,^14^. Hypothalamic dysfunction is sufficient to induce obesity and diabetes in genetic mouse models with neuronal ablation of insulin signaling^15^. These findings indicate that hypothalamic dysfunction plays an important role in altered energy metabolism.

UA has been shown to have pro-inflammatory activity. It induces NADP-oxidase activation by activating redox-dependent pro-inflammatory signaling in cultured adipocytes^13^. Additionally, UA upregulates the expression of *Crp*, a marker of inflammation, in human vascular smooth muscle cells and endothelial cells^16^. However, whether it is neuroprotective or neurotoxic in the central nervous system remains controversial^17^,^18^. Few studies have examined the relationship between UA and hypothalamic inflammation.

NF-κB is a pro-inflammatory master switch that controls the production of inflammatory markers and mediators. NF-κB activation induced by UA has been reported in several diseases. For example, UA induces renal inflammation in gouty nephropathy via the activation of tubular NF-κB signaling^19^. Soluble UA induces inflammatory pathways by activating p38 MAPK and NF-κB in rat vascular smooth muscle cells^20^. Recent studies have revealed that the pro-inflammatory NF-κB pathway mediates hypothalamic inflammation to cause metabolic syndrome^12^,^21^. It is of note that in addition to being an inflammatory regulator, IKKβ/NF-κB controls cell survival, growth, apoptosis and differentiation in a cell-specific manner^22^. Specific suppression of NF-κB signaling broadly across the brain, locally within the mediobasal hypothalamus, or specifically in hypothalamic AgRP neurons significantly protects against metabolic disorders^12^.

In the present study, we found that serum UA can pass through the blood-brain barrier (BBB) and act as a potent inflammatory stimulus, leading to NF-κB activation as well as the accumulation of gliosis in the hypothalamus, both in animal models and in humans.

## Results

### Systemic hyperuricemia induces hypothalamic inflammation in rodents

**Figure 1:**
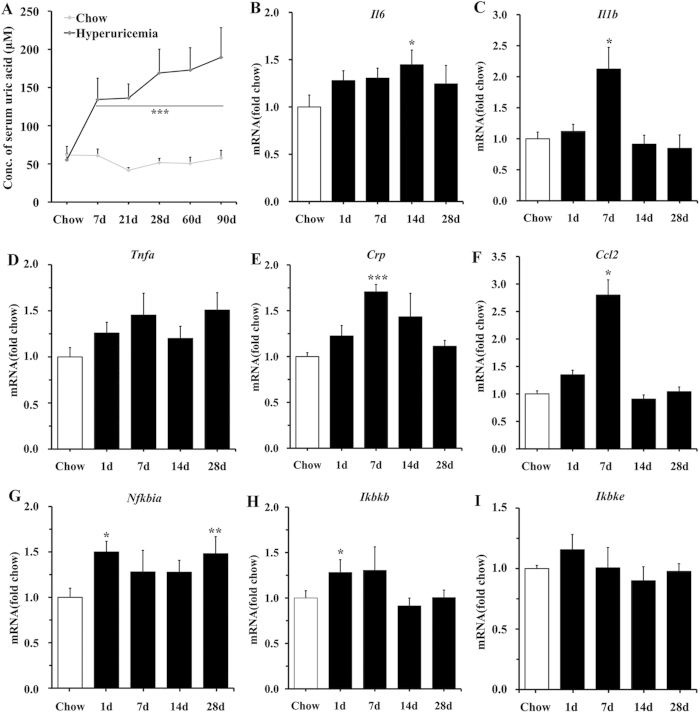
Systemic hyperuricemia induces hypothalamic inflammation in rodents. (**A**) Time course of serum UA concentration after the onset of HUAD feeding (n = 6 rats per group; Avg. = average). (**B–I**) Time course of the induction of mRNA expression of inflammatory mediators, including pro-inflammatory cytokines (*Il6*, *Il1b*, *Tnfa, Crp, Ccl2*) and NF-κB signaling (*Nfkbia*, *Ikbkb*, *Ikbke*) in the hypothalamus of rats fed chow or a HUAD for up to 28 days (n = 6 rats per group). All mRNA species were quantified relative to *Gapdh* housekeeping gene expression and are presented as fold changes relative to chow-fed controls [fold chow]. All displayed values are the mean ± SEM. *P < 0.05; **P < 0.01; ***P < 0.001 versus chow-fed control.

To investigate the effect of UA on the hypothalamus, we applied a previously reported strategy to generate a hyperuricemia model. Rats were fed a high-UA diet (HUAD) containing 2% oxonic acid (OA) and 2% UA for 3 months^23^,^24^. OA can inhibit UA to metabolize into allantoin in rodents^6^. But it’s a partial inhibitor with a short half-life and rats treated with OA alone develop mild hyperuricemia, we added UA as a supplement. Serum UA concentrations in rats fed a HUAD for 28 days were increased relative to control rats fed standard chow and were approximately 3-fold higher than that in control rats at the end of 3 months (Fig. 1A), indicating that the hyperuricemic rat model was developed successfully.

Hypothalamic inflammation is an early event in the development of metabolic disorders and is evident in rodents within 1–3 days of high-fat-diet onset^25^. Using quantitative real-time PCR (qRT-PCR), we analyzed the transcriptional levels of inflammatory genes in the hypothalamus. We found that the gene expression profile exhibited a complex “on-off” pattern, which showed upregulation in *Il1b*, *Tnfa*, and *Ikbkb* mRNA within the first 7 days of HUAD administration, followed by a decline to baseline or to a slightly elevated level from days 14 to 28 (Fig. 1C,D,H). The expression levels of *Il6* and *Nfkbia* were elevated even on the first day of the HUAD and increased by approximately 50% within 28 days (Fig. 1B,G). These results showed that HUAD could induce hypothalamic inflammatory gene expression.

In addition to the hypothalamus, we also detected the pro-inflammatory cytokines expression in the prefrontal cortex and hippocampus. The results showed that pro-inflammatory cytokines increased in the hippocampus after 3 months of HUAD administration. It has been reported that obesity-induced diabetes is associated with hippocampal chronic inflammation and is considered a risk factor for neurodegeneration^26^,^27^. We hypothesize that UA may be associated with memory impairments. However, because we aimed to investigate the effect of UA on metabolism regulation, we mainly focused on the hypothalamus in this study.

### Markers of gliosis during serum UA elevation

**Figure 2:**
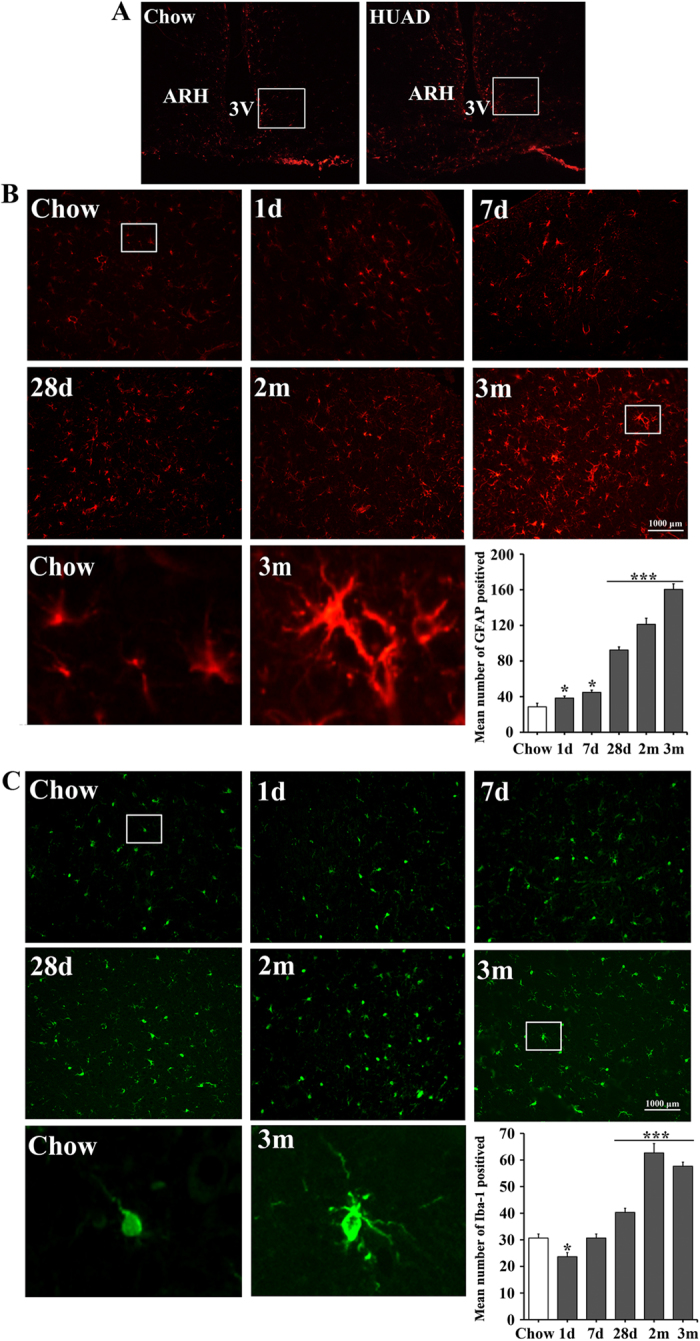
Immunofluorescence analysis of UA-induced astrocyte and microglial accumulation in the rat ARC. (**A**) Hypothalamic sections of the rats fed a standard chow (left) or a HUAD (right) were immunostained for GFAP. The image is displayed at × 100 original magnification, and the white box indicates the region acquired for the quantification of ARC astrocyte and microglia numbers. (**B,C**) Immunofluorescence detection of the astrocytic marker GFAP protein (**B**) and of the microglial marker Iba-1 (**C**) in coronal sections of the rat hypothalamus (4 μm) from rats fed either chow or a HUAD for 1 day, 7 days, 28 days, 2 months and 3 months. The low-magnification image is × 200 original magnification, which was used for the quantification of ARC astrocyte and microglia numbers. The white box indicates the region presented in the magnification below, which was used to show the activated morphology of the cells. The mean number of ARC astrocytes and microglia in rats fed either chow or a HUAD were quantified in the whole region from the low-magnification view displayed on the lower right (means ± SEM; n = 6 rats per group). *P < 0.05; **P < 0.01; ***P < 0.001 versus chow-fed control.

The activation, recruitment, and proliferation of microglia and astrocytes, collectively termed “reactive gliosis”, is a hallmark of the brain’s response to neuronal injury^25^. The effect of UA on astroglial responses in rats was assessed by GFAP immunostaining. As expected, GFAP-positive astrocytes were scattered throughout the arcuate nucleus (ARC) of rats (Fig. 2A). Within 28 days of HUAD administration, however, the intensity of GFAP staining in the ARC increased by approximately 2-fold and remained at this high intensity over the next 2 months (Fig. 2B; quantified on the lower right), suggesting an effect of HUAD in promoting astrocyte accumulation in this brain area. Furthermore, the astrocytes became increasingly enlarged, indicative of a more active morphology. Using immunofluorescence to detect the microglia-specific cytoplasmic marker Iba-1, we found that the number of microglia clearly increased in the ARC over the duration of HUAD exposure compared to those in the chow-fed controls (Fig. 2C). Concomitantly, microglia increased in size and adopted a more activated morphology. Collectively, these results showed that the rapid onset of hypothalamic inflammation induced by a HUAD was accompanied by robust glial responses in the MBH.

### UA passes through the BBB and acts as an inflammatory stimulus in the hypothalamus

**Figure 3:**
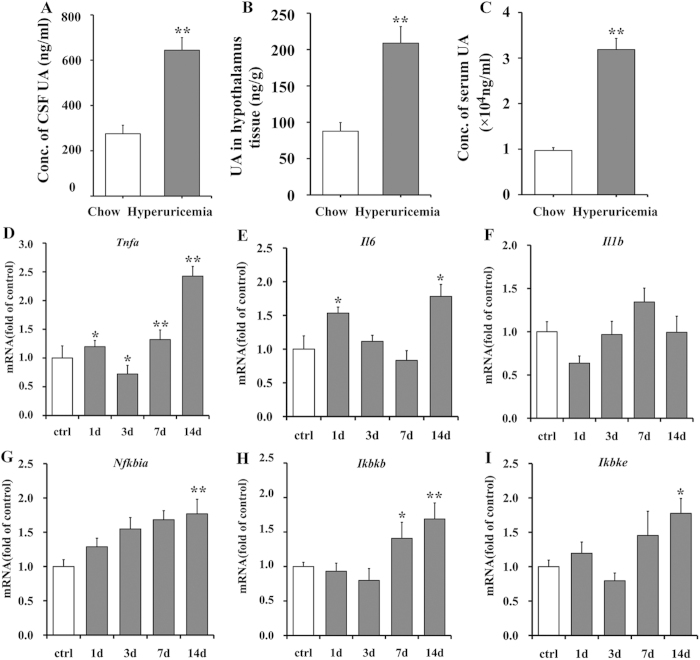
Icv administration of UA induces hypothalamic inflammation. (**A–C**) UA concentration in the CSF (**A**), the amount of UA in hypothalamic tissue (**B**), and fasting serum UA concentration (**C**) were determined in rats fed chow or a HUAD for up to 3 months. (**D–I**) Time course of the induction of mRNA expression of the inflammatory mediators *Tnfa* (**D**), *Il6* (**E**), *Il1b* (**F**), *Nfkbia* (**G**), *Ikbkb* (**H**), and *Ikbke* (**I**) in the hypothalamus of the rats that received icv administration of saline or 3000 ng/ml UA for up to 2 weeks (n = 6 rats per group). All mRNA species were quantified relative to *Gapdh* housekeeping gene expression and are presented as fold changes relative to saline controls [fold of control]. All displayed values are the mean ± SEM. *P < 0.05; **P < 0.01; ***P < 0.001 versus normal saline control.

To investigate whether UA could pass through the BBB and play a role on the brain, we assessed UA levels in the cerebrospinal fluid (CSF) and in hypothalamic tissue using liquid chromatography tandem mass spectrometry. We found that the average UA concentration in the CSF of HUAD-fed rats was 644.25 ng/ml, which was 1.34-fold higher than the concentrations of chow-fed control rats (Fig. 3A). The amount of UA in the hypothalamic tissue of HUAD-fed rats was 208.9 ng/g, whereas it was 87.7 ng/g in chow-fed controls (Fig. 3B). The serum UA concentrations in the HUAD and control groups were 31.86 and 9.69 μg/ml, respectively (Fig. 3C). These data indicated that UA could pass through the BBB and deposit in hypothalamic tissue. Additionally, the amount of UA in both the hypothalamus and CSF of HUAD-fed rats was significantly elevated.

To determine the direct effect of UA in inducing pro-inflammatory cytokines in the hypothalamus, rats were intracerebroventricularly (icv) cannulated and perfused continuously with UA. It has been reported that the average volume of ventricular CSF (lateral, third, and fourth ventricles) in the rat is 7–34 μl, the total cranial CSF volume is 200–440 μl, and the CSF flow rate within the ventricular system is 2.5–3.7 μl /min^28^,^29^. According to these values, the dose of UA used in this study was 3000 ng/ml, with a volume of 10 μl and a rate of 1 μl/min. As expected, 14 days of icv UA treatment produced a significant increase in the expression of pro-inflammatory cytokines in the hypothalamus (Fig. 3D–I), some of which increased as early as the first day. These results suggested that UA can act directly on the hypothalamus and induce hypothalamic inflammation.

### Changes in gliosis upon UA stimulation

**Figure 4:**
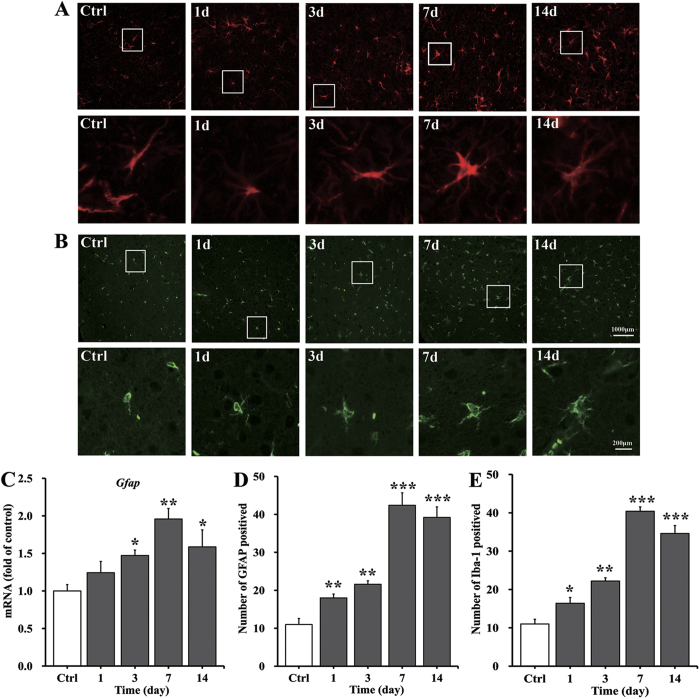
Fluorescence immunohistochemical analysis of the accumulation of astrocytes and microglia in the rat ARC induced by icv UA perfusion. (**A,B**) Immunofluorescence detection of the astrocyte marker GFAP protein (**A**) and of the microglial marker Iba-1 (**B**) in coronal sections of the rat hypothalamus (4 μm) from rats treated with icv saline as a control or with UA (3000 ng/ml, 10 μl) for 1, 3, 7 and 14 days. The low-magnification image is × 400 original magnification, which was used for the quantification of ARC astrocyte and microglia numbers. The white box indicates the region presented in the magnification below, which shows the activated morphology of the cells. (**C**) The astrocyte marker *Gfap* was quantified by qRT-PCR in rats treated with icv saline or UA (3000 ng/ml, 10 μl) perfusion for 1, 3, 7 and 14 days (n = 6 rats per group). The mRNA level of *Gfap* was quantified relative to *Gapdh* housekeeping gene expression and is presented as a fold change relative to the saline control [fold of control]. (**D,E**) The mean number of ARC astrocytes (**D**) and microglia (**E**) in the rats with icv administration of saline or UA were quantified in the whole region using a low-magnification view (n = 6 rats per group). All displayed values are the mean ± SEM. *P < 0.05; **P < 0.01; ***P < 0.001 versus normal saline control.

To detect the rapid onset of gliosis in response to UA, the gene expression of *Gfap* in the hypothalamus was assessed. We found that hypothalamic *Gfap* mRNA levels increased by 47% after UA icv perfusion for 3 days and were elevated by 96% at day 7. At day 14, a slight elevation of 59% was observed (Fig. 4C). The number of astrocytes in the ARC increased almost 3-fold (Fig. 4A; quantified in Fig. 4D); moreover, we observed through visual evaluation that the size of astrocytes became increasingly larger, indicative of a more active morphology (Fig. 4A). In addition, the effect of UA on microglia was assessed by Iba-1 immunostaining. The number of microglia increased significantly in the ARC on the first day of UA icv perfusion, remained elevated throughout the 2-week period, and increased up to 2- to 3-fold higher than the control at the end of the experiment (Fig. 4B; quantified in Fig. 4E).

### NF-κB is the main link between UA and hypothalamic inflammation

**Figure 5:**
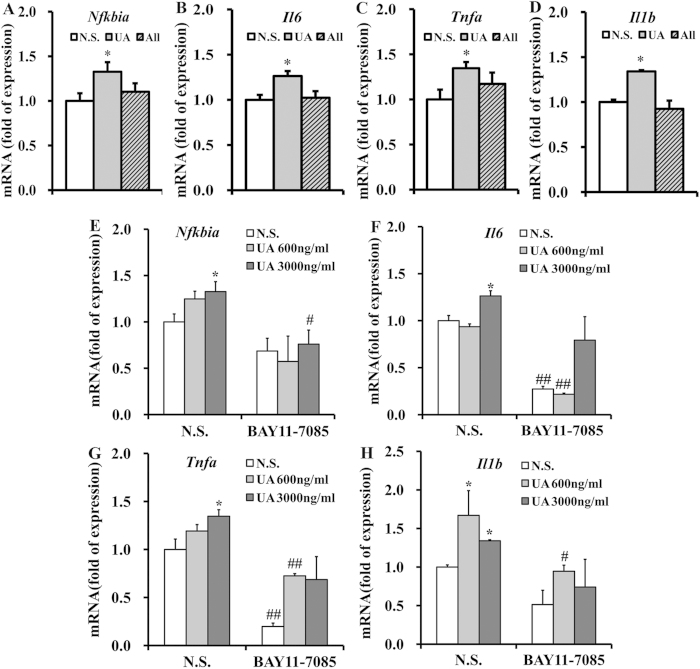
Inhibition of NF-Κb protects mice against the hypothalamic inflammation induced by UA. (**A–D**) Expression of *Nfkbia* (**A**), *Il6* (**B**), *Tnfa* (**C**), and *Il1b* (**D**) were determined by qRT-PCR in the hypothalamus of the mice that were icv injected with normal saline (N.S.), UA (3000 ng/ml, 2 μl) or allantoin (3000 ng/ml, 2 μl) for 2 weeks (n = 6 mice per group). (E–H) Expression of *Nfkbia* (**E**), *Il6* (**F**), *Tnfa* (**G**), and *Il1b* (**H**) were determined by qRT-PCR in the hypothalamus of the mice that were icv injected with normal saline (N.S.) or UA (600 ng/ml or 3000 ng/ml, 2 μl), with or without icv injection of BAY11-7085 (n = 6 mice per group). All displayed values are the mean ± SEM. *P < 0.05; **P < 0.01 versus saline control; ^#^*P* < 0.05; ^##^*P* < 0.01 versus without BAY11-7085 administration.

To further verify that hypothalamic inflammation was induced by the pathogenic effect of UA rather than transient stimulation, C57BL/6J mice were icv cannulated and perfused with 3000 ng/ml UA or an equal dose of allantoin, a metabolite of UA in rodents. After 2 weeks of administration, the expression of hypothalamic pro-inflammatory cytokines and *Nfkbia* was determined. We found that UA markedly increased the mRNA levels of the pro-inflammatory cytokines and *Nfkbia* (Fig. 5A–D). However, allantoin did not exhibit such an effect (Fig. 5A–D). These results indicated that the pathogenic effect of UA but not transient UA stimulation may contribute to hypothalamic inflammation.

NF-κB is a critical mediator of nutritionally induced inflammation and metabolic dysfunctions^30^,^31^, we hypothesized that NF-κB may link UA to hypothalamic inflammation. To explore this hypothesis, BAY11-7085, a specific NF-κB inhibitor, was administered into the lateral ventricle 30 min before UA icv administration in C57BL/6 J mice^32^. Importantly, BAY11-7085 markedly inhibited the UA-induced upregulation of pro-inflammatory cytokines and *Nfkbia* (Fig. 5E–H), suggesting that hypothalamic NF-κB activation may be an important signaling event linking UA with hypothalamic inflammation.

### UA induces NF-κB activation in neurons *in vitro*

**Figure 6:**
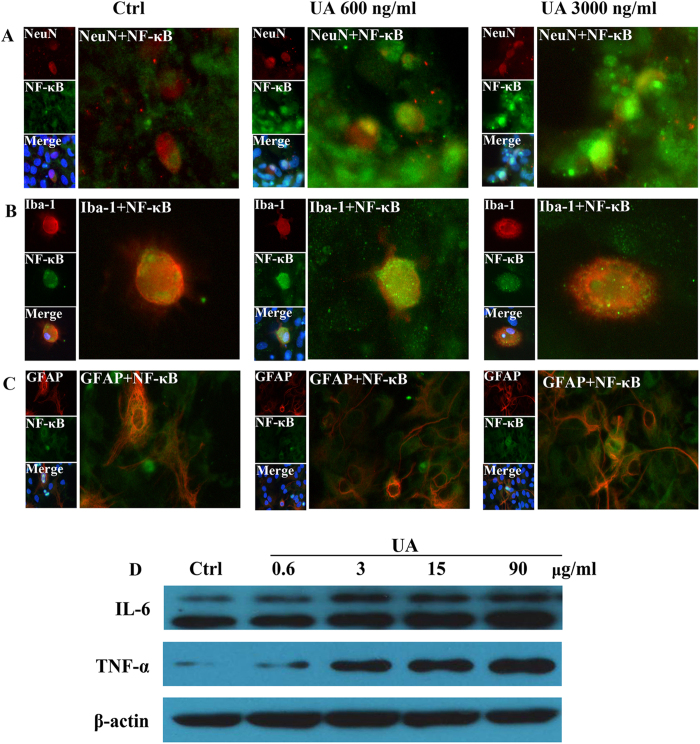
Immunofluorescence analysis of NF-κB activation in primary cultured neurons and glia cells. (**A**–**C**) Hypothalamic primary cultures were immunostained for NeuN and RelA (**A**), Iba-1 and RelA (**B**), and GFAP and RelA (**C**) ( × 400) after 24 h exposure to 600 ng/ml or 3000 ng/ml UA. RelA was used for the reporting of NF-κB. Hoechst nuclear staining revealed all cells in the section. (**D**) SH-SY5Y cells were treated with normal saline (Ctrl) or UA (0.6, 3, 15, 90 μg/ml) for 48 h, and then the protein expression of TNF-α, IL-6 and β-actin were analyzed by western blotting. The gels were run under the same experimental conditions and data typical of 3 independent experiments are shown. The full-length blots are presented in Supplementary Figure S2.

Our findings described above confirmed that hypothalamic inflammation and gliosis occurred as early as the first day of the hyperuricemia model in rats. To further demonstrate the UA-mediated hypothalamic inflammation, immunostaining for NeuN, GFAP, Iba-1, and the p65/RelA subunit of NF-κB was performed in primary cultures of hypothalamic cells. We observed p65/RelA activation in neurons after exposure to 600 and 3000 ng/ml UA for 24 h (Fig. 6A), which was characterized by p65/RelA translocation to the nucleus. However, p65/RelA was barely activated in glial cells exposed to UA (Fig. 6B,C). To further study the potential role of UA in inducing inflammation, western blotting was carried out to detect the expression of pro-inflammatory cytokines in cultured SH-SY5Y human neuroblastoma cells, which naturally express insulin and leptin receptors. Consistently, TNF-α and IL-6 expression was also significantly increased in SH-SY5Y cells exposed to UA for 48 h (Fig. 6D).

### Magnetic resonance imaging (MRI)-based quantitative assessment of hypothalamic gliosis in hyperuricemia rodents and humans

Gliosis is a well-characterized neural tissue response to injury from inflammatory insults^33^. We showed that consuming a HUAD induced inflammation associated with gliosis in the hypothalamus of rats. To histologically confirm the gliosis induced by the HUAD, MRI was used to detect gliotic changes quantitatively.

**Figure 7:**
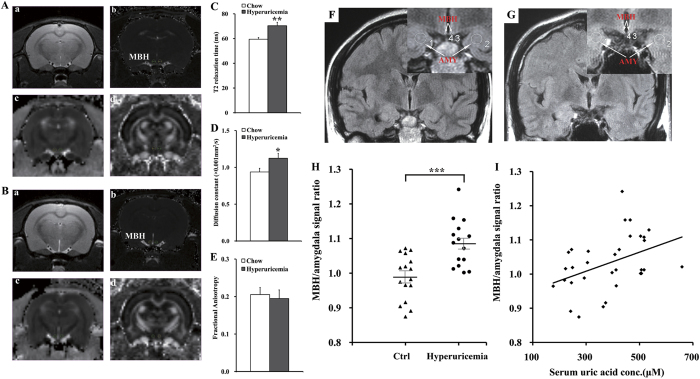
MRI-based quantitative assessment of hypothalamic gliosis in hyperuricemia rodents and humans. (**A,B**) Regions of interest (circles) and representative images of the normal rats (**A**) and hyperuricemia rats (**B**). a. High-resolution, 2-dimensional rapid acquisition; b. T2 map generated from a multiecho sequence; c. DTI for tensor trace measurement; d. DTI for fractional anisotropy. (**C–E**) Results of multiparametric quantitative assessment in hyperuricemia rats and chow-fed controls, including T2 relaxation time (**C**) (ms = millisecond), tensor trace (**D**) and fractional anisotropy (**E**) (n = 6 rats per group). (**F,G**) Representative coronal T2 FES FLAIR images through the hypothalamus in a normal serum UA subject (**F**) and a hyperuricemia subject (**G**). Insets show the placement of ROIs (white circles) in the mediobasal hypothalamus (MBH) and amygdala (AMY). (**H**) The average signal ratio on both sides that signal intensity within ROIs placed in the MBH versus ROIs in adjacent amygdala tissue was used for quantitative assessment in normal serum UA subjects (n = 16 subjects) and hyperuricemia subjects (n = 15 subjects). (I) Correlation of serum UA concentration with MBH hyperintensity, as measured by average MBH/amygdala signal ratio on both sides (n = 31 subjects; *r* = 0.41). All displayed values are the mean ± SEM. *P < 0.05; **P < 0.01; ***P < 0.001 versus control.

Regions of interest (ROIs) and example images from the 2-dimensional sequence, T2 parametric map, and DTI-EPI sequence are shown (Fig. 7A,B). There was a significant difference in T2 relaxation time in the hypothalamic ROIs between hyperuricemia rats and chow-fed rats, indicating that UA could significantly increase T2 relaxation times (Fig. 7C). Moreover, the effect of lengthening T2 relaxation time in the MBH persisted when the left and right MBH were analyzed separately in the model (left *P* < 0.01; right *P* < 0.05); however, laterality (right vs. left) had no effect (*P* = 0.13). Using diffusion tensor imaging (DTI), values were compared for fractional anisotropy and tensor trace assessments. The tensor trace showed a pattern similar to the T2 relaxation times, confirming that inflammation occurred (Fig. 7D); however, laterality (right vs. left) showed no effect (*P* = 0.41). Nevertheless, there was no significant difference in fractional anisotropy (Fig. 7E). These results confirmed that gliosis occurred in the hypothalamus of the hyperuricemia rats.

We then investigated whether MBH gliosis could also occur in hyperuricemia subjects. We analyzed the data obtained from 15 hyperuricemia subjects with MRI examinations and from 16 normal subjects. The concentration of serum UA in these hyperuricemia subjects ranged from 424 to 659 μM for men and from 397 to 518 μM for women, whereas the average concentration was 293.1 μM in normal subjects. We performed an initial inspection of the MBH for hyperintense signal in T2 coronal sections, as performed in the rodent study described above. Hyperintense signal in the MBH is a characteristic finding of gliosis in numerous inflammatory, ischemic, and degenerative neural disorders in clinical^34^. To detect gliotic changes below the visual detection threshold and to eliminate the influence of changes in background between different batches of imaging, ratios were created to compare the mean signal intensity between ROIs placed in the MBH and ROIs in adjacent amygdala tissue. ROIs are shown by white circles and arrows in the representative images from a normal subject and a hyperuricemia subject (Fig. 7F,G). Very importantly, in group comparisons, the right and left MBH/amygdala mean signal intensity was significantly higher among hyperuricemia subjects (1.083 ± 0.019) than that in normal UA subjects (0.988 ± 0.016) (Fig. 7H). Collectively, our retrospective analysis suggested that hyperuricemia in humans is very likely associated with gliosis in the MBH (Fig. 7I), which is a well-characterized neural tissue response to inflammatory insults^33^.

### UA leads to dyslipidemia and glucose intolerance

**Figure 8:**
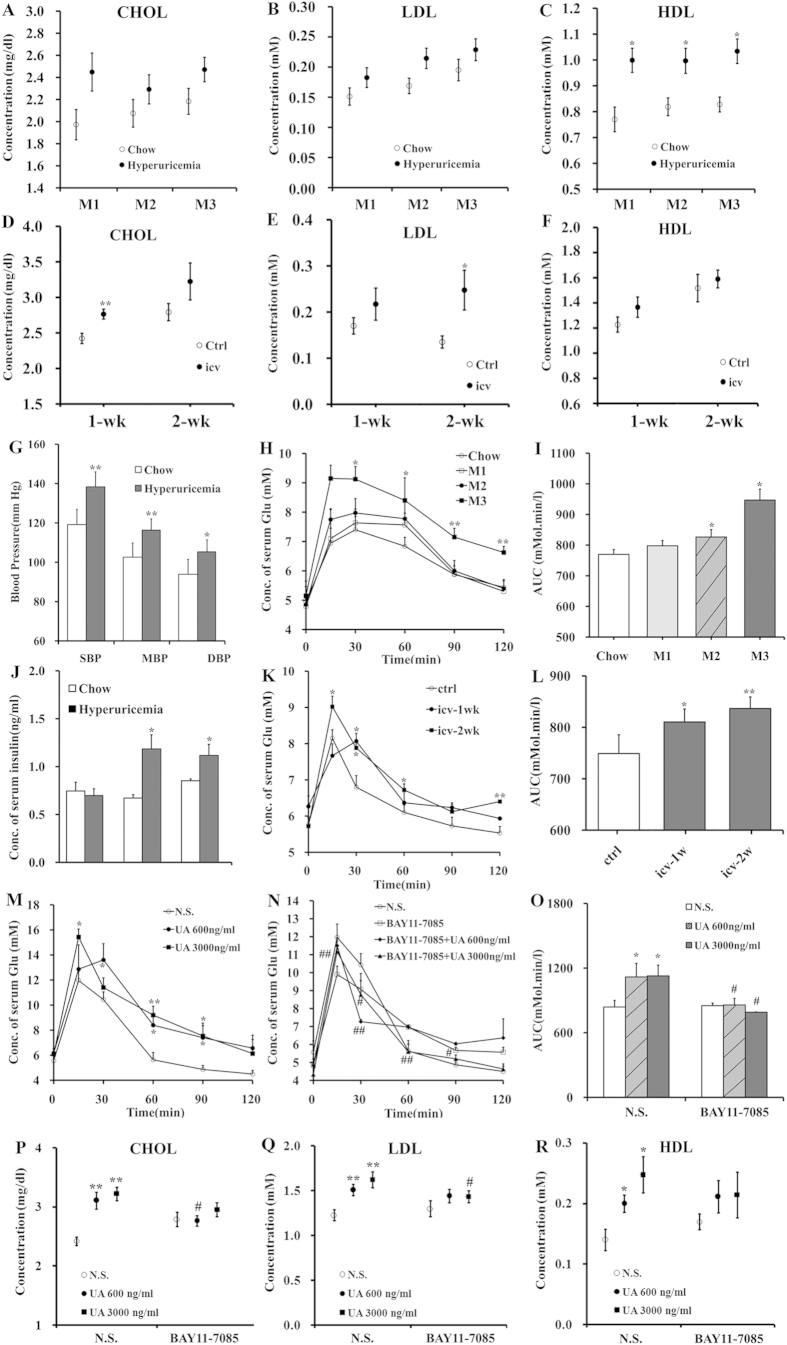
UA leads to dyslipidemia and glucose intolerance mediated by NF-κB. (**A–C**) Time course of serum biochemical parameters after the onset of HUAD feeding (n = 6 rats per group). (**D–F**) Serum biochemical parameters of the rats that were icv administered saline or UA for 1 and 2 weeks (n = 6 rats per group). (**G–I**) Blood pressure measurements (**G**) and an OGTT were performed in rats fed chow or a HUAD for 1, 2, and 3 months; glucose concentrations are plotted versus time (**H**) and as AUC (**I**, per 120 min) (n = 6 rats per group). (**J**) Quantification of fasting serum insulin concentration in rats fed chow or a HUAD for 1, 2, and 3 months (n = 6 rats per group). (**K**,**L**) An OGTT was performed in the rats that received icv administration of saline or UA (3000 ng/ml, 1 μl/min, 10 μl) for 1 and 2 weeks (icv-1w and icv-2w). The test was performed 24 h after the last dose of UA, and glucose concentrations are plotted versus time (**K**) and as AUC (**L**, per 120 min) (n = 6 rats per group). (**M–O**) OGTTs were performed in mice with icv injection of normal saline (N.S.) or UA (600 ng/ml or 3000 ng/ml, 1 μl/min, 2 μl), with (**N**) or without (**M**) icv injection of BAY11-7085 (250 mM, 1 μl/min, 2 μl) 30 min prior to the icv UA administration for 14 consecutive days. The test was performed 8 h after the last dose of UA, and glucose concentrations are plotted versus time (**M,N**) and as AUC (**O**, per 120 min) (n = 6 mice per group). (**P–R**) Serum biochemical parameters of the mice that were icv administered saline or UA, with or without icv injection of BAY11-7085 (n = 6 mice per group). All displayed values are the mean ± SEM. *P < 0.05; **P < 0.01; ***P < 0.001 versus control; #P < 0.05; ##P < 0.01 versus without BAY11-7085 administration.

The hypothalamus is the nerve center responsible for metabolism. To track UA in relation to lipid and glucose metabolism, we measured several biochemical parameters in serum collected from the HUAD-fed rats (Fig. 8A–C) and UA icv-injected rats (Fig. 8D–F). Interestingly, the rats treated with these two UA administration approaches showed dyslipidemia. The blood pressure of the rats subjected to 3 months of a HUAD was somewhat increased (Fig. 8G). We then performed an oral glucose tolerance test (OGTT) and a fasting serum insulin detection test at various time points^7^,^35^. We found that HUAD-fed rats exhibited a significantly higher blood glucose concentration (Fig. 8H,I) as well as an increased level of serum insulin (Fig. 8J) than chow-fed control rats. The same was true for UA icv-injected rats. In the OGTT, the glucose area under the curve (AUC) showed an 8% and 12% increase in the rats receiving icv UA perfusion for 1 and 2 weeks, respectively (Fig. 8K,L).

Moreover, glucose intolerance also occurred in the mice receiving icv UA perfusion, raising the possibility of a common phenomenon of UA-induced glucose metabolic disorders across species (Fig. 8M). Importantly, the inhibition of NF-κB by icv injection of BAY11-7085 protected mice against UA-induced glucose intolerance (Fig. 8N); moreover, the glucose AUC in the OGTT almost recovered to normal (Fig. 8O). The UA-induced elevation of CHOL, LDL, and HDL was also ameliorated by NF-κB inhibition (Fig. 8P–R). These results suggested that NF-κB plays an important role in UA-induced metabolic disorders.

To investigate the events linking UA-induced hypothalamic inflammation to metabolic disorders, we measured mRNA levels of the relevant hypothalamic neuroendocrine factors involved in the control of feeding, glucose levels and thermogenesis^36^. Importantly, we found an “on-off” pattern in the gene expression; mRNA levels of *Agrp*, *Pomc*, *Crh*, *Trh*, *Ghrh*, and *Gnrh* were elevated in the hypothalamus within the first 7 days of HUAD administration, followed by a decline to almost 50% of the control levels from days 14 to 28 (Supplementary Fig. S1A,B). And two of the neuroendocrine factors, *Crh* and *Trh*, which are important for thermogenic function, decreased in the rats with icv UA administration (Supplementary Fig. S1C). Because the transcriptional integration of NF-κB, c-Jun and PKC pathways seems to account for the downregulation of hypothalamic neuroendocrine factors, we then assessed the mRNA levels of the *c-fos*, *c-jun*, *Prkca*, *Prkcd*, and *Prkcq* genes in the hypothalamus of rats that received a HUAD for 14 and 28 days (Supplementary Fig. S1D) or that received icv UA perfusion for 14 days (Supplementary Fig. S1E). Interestingly, we found that *Prkcd*, the gene that encodes PKC-δ, changed significantly, suggesting that UA activates hypothalamic NF-κB signaling and causes neuroendocrine disorders at least partially through PKC-δ, eventually leading to systemic metabolic disorders.

## Discussion

In the present study, pro-inflammatory cytokine gene expression displayed a “on-off” pattern in response to UA, and an increase in pro-inflammatory cytokines and gliosis in the hypothalamus occurred during the early phase of UA stimulation. We thus hypothesize that the rapid onset of MBH inflammation could be a manifestation of neuronal injury that then triggers reactive gliosis involving microglial and astroglial cell populations, both of which are thought to limit the extent of inflammation and neuron loss in brain injury^37^. The transient response of glial cells to UA suggests that neuroprotective responses could be mounted to limit or reverse the injury during its initial phases and cause the expression of pro-inflammatory markers to return to basal levels in the MBH. However, with sustained exposure to UA, the capacity of supportive glial cells to control the damage is exceeded, such that chronic inflammation and reactive gliosis are generated in the hypothalamus. Our findings in primary hypothalamic neural cells also showed that NF-κB was activated in neurons but not in glial cells after UA exposure. Therefore, it is reasonable to conjecture that glial cells play an important neuroprotective role under conditions in which the initial UA-induced insult is likely directed at neurons rather than glial cells. Our findings are supported by similar observations in demyelinating or neurodegenerative disease^38^,^39^.

Several mechanisms have been reported to explain overnutrition-induced inflammation, including the activation of TLR4, serine/threonine kinases and endoplasmic reticulum stress^40^. In the present study, the gene expression of *Ikbkb*, *Ikbke*, and *Nfkbia* increased significantly after UA stimulation, suggesting that UA-induced inflammation may be mediated by the activation of the IKK/NF-κB pathway. In fact, previous studies have demonstrated that IKKβ/NF-κB activation in hypothalamic neurons is a general cause of metabolic abnormalities due to overnutrition^41^. Conversely, ablation of IKKβ in hypothalamic AgRP and POMC neurons significantly protects against glucose intolerance^12^. In fact, in addition to its role as an inflammatory regulator, NF-κB signaling also controls cell survival, apoptosis and synaptic plasticity^42^. For example, p65 regulates the proliferation of adult neural stem cells through the NF-κB target genes c-myc and cyclin D1^43^,^44^. In the mature nervous system, NF-κB is activated in synapses in response to excitatory synaptic transmission and may play a pivotal role in processes such as learning and memory^45^.

Whether IKK/NF-κB signaling is activated in glial cells in conditions of overnutrition has been heavily debated until now. Some studies have shown that the hypothalamic dysregulation induced by overnutrition involves a neuron-specific program through IKKβ/NF-κB and that inflammatory cytokines do not have strong effects in non-neuronal cells^31^. However, others have shown that in conditions of chronic inflammation induced by overnutrition and senescence, NF-κB is activated in microglia, which are regarded as the macrophage-like immune cells of the brain. Moreover, pro-inflammatory cytokines, such as TNF-α and IL-6, released from microglia work on appetite-controlling neurons to further activate NF-κB^27^. In the primary hypothalamic culture experiments performed in the present study, NF-κB was mainly activated in neurons in response to UA and not in glial cells, suggesting that a neuron-specific program involving IKK/NF-κB signaling pathway plays a role.

In recent years, multiparametric high-field MRI approaches have been used to assess and quantify gliosis in the MBH, based on the techniques of T2 relaxation time or DTI^34^. In particular, T2 relaxation time is significantly correlated with histopathological gliosis and is considered a promising quantitative radiological marker of gliosis in the MBH, despite its minute size^33^,^34^. In our study, T2 relaxation times were significantly different in the gliotic MBH of HUAD-fed rats, which was characterized by an increased density of both astrocytes and microglia. We extended the finding of the high MBH T2 signal by associating it with high serum UA levels in humans. Importantly, through a retrospective analysis of T2 MRI obtained in a cohort of human subjects undergoing clinical examination, we found that the intensity of the T2 signal (hyperintensity is an indicator of gliosis) was significantly increased in the human MBH in individuals with hyperuricemia. This finding does not constitute definitive proof of increased gliosis because edema, infection, and tumors could have a similar appearance. However, subjects with preexisting evidence of neurological abnormalities were excluded from the study, and these alternative explanations for increased T2 signal intensity are unlikely. Because MRI is a safe and established tool for human brain imaging, our study suggests that MRI-based techniques have translational value because they can be used to make comparable measurements of gliosis in the MBH of humans. MRI may be a potential technique for the prediction of metabolic diseases because hypothalamic gliosis is an early event that precedes dysfunction in peripheral tissues.

We found that rats exhibited dyslipidemia and glucose intolerance during UA stimulation and that the inhibition of hypothalamic inflammation by an NF-κB inhibitor remarkably ameliorated glucose intolerance. This evidence suggests that the inflammation induced by UA in the hypothalamus could be one of the potential causes of metabolic disorders. This notion is supported by previous studies that evaluated the isolated effect of low-grade hypothalamic inflammation on peripheral insulin action. Lean rats with icv administration of a low dose of TNF-α exhibit impairment of leptin’s anorexigenic effects, hyperglycemia, and impairment of insulin signal transduction in liver and muscle^14^,^46^. Additionally, icv injection of saturated fatty acids induces an increase in TNF-α expression and inflammatory signal transduction through the activation of TLR4 and PKCτ in the hypothalamus^47^,^48^. In fact, a growing number of studies has indicated that the key mechanisms of dyslipidemia and glucose intolerance may originate in the brain, especially in the hypothalamus, because multiple hypothalamic changes occur prior to substantial diabetes and obesity^25^,^49^. Therefore, we speculate that some nutrients in the diet or abnormal levels of endogenous substances, such as UA in our study, may stimulate the hypothalamus to produce inflammation directly, leading to the changes in various peripheral tissues.

In summary, the consumption of a HUAD induces hypothalamic inflammation and reactive gliosis in both rodents and humans. Experimental and clinical data indicate that hypothalamic inflammation mediated by NF-κB signaling may be implicated in the pathogenesis of metabolic disorders induced by UA. However, there is a limitation in our study. In the retrospective study in humans, we only compared mediobasal hypothalamus MRI signal intensity of hyperuricemia group and the normal group. But due to time constraints, we did not track the prognosis of these subjects, which is likely to further illustrate the viewpoint of this study.

## Methods

### Preparation of reagents

UA used in icv administration *in vivo* or in cell culture was prepared as follows: UA was dissolved in saline, sonicated for 30 min, and filtered with a 0.22 μm membrane filter, followed by observation under a microscope to confirm the absence of UA crystals.

### Animal studies

Weight-matched male Wistar rats (300–350 g) and male C57BL/6J mice (7–8 weeks old) were purchased from Beijing Animal Center (Beijing, China). All of the animals were housed individually in a specific pathogen-free environment, maintained in a temperature-controlled room with a 12-hour-light/12-hour-dark cycle, and provided with *ad libitum* access to water.

To establish the hyperuricemia model, the rats were provided with a HUAD containing 2% OA and 2% UA for periods ranging from 1 day to 3 months^23^,^24^. The animals were anaesthetized with sodium pentobarbital before necropsy. All of the animal protocols were reviewed and approved by the Experimental Animal Ethics Committee of Sichuan University (Chengdu, China)^50^,^51^, and the methods were carried out in accordance with the approved guidelines.

### Icv cannulation and icv administration

Wistar rats and C57BL/6J mice were placed in a stereotaxic apparatus (Ruiwode, Shenzhen, China) according to a previously described method^52^. A stainless steel guide cannula was stereotaxically implanted into the lateral cerebral ventricle of the rats (1.5 mm lateral and 0.8 mm posterior to bregma, 4.0 mm ventral to the surface of the skull). After cannulation, the rats were treated with ampicillin sodium (0.2 g/ml, 600 μl/kg, intramuscular, twice daily) for 4 days. After a 3-day recovery period, the icv-cannulated rats were treated daily with 10 μl UA (3000 ng/ml, 1 μl/min) for 1, 3, 7 or 14 days.

The coordinates (relative to bregma) for icv cannula implantation in mice were 1.0 mm lateral, -0.42 mm anteroposterior, and 2.2 mm deep^53^. After cannulation, the mice were treated the same as the rats (described above). After 4 days of antimicrobial therapy and 3 days of recovery, the icv-cannulated animals were administered 2 μl normal saline, UA (600 ng/ml and 3000 ng/ml) or allantoin (3000 ng/ml) daily for 14 days. To inhibit NF-κB activation in the hypothalamus, 2 μl BAY11-7085 (500 nmol) was icv injected 30 min before the injection of UA^32^. The infusion rate for normal saline, UA, allantoin, and BAY11-7085 was approximately 1 μl/min.

### Retrospective study of brain MRI in humans

We performed a retrospective cohort study to search for radiological evidence of MBH gliosis and to correlate our findings with hyperuricemia. Magnetic resonance brain examinations that used pituitary or epilepsy imaging protocols with coronal T2 FES FLAIR sequences and that were performed between 3/1/2014 and 6/20/2014 at West China Hospital of Sichuan University were reviewed for 2 inclusion criteria: the availability of high quality coronal views of the hypothalamus and the absence of clinical abnormalities that might confound interpretation (e.g., hypothalamic-pituitary axis disease). For the 51 subjects that met our inclusion criteria, we reviewed electronic medical records to determine serum UA concentration, gender, age, body weight, and final diagnosis. Exclusion criteria were as follows: absence of serum biochemical analysis for UA; age of less than 16 or more than 70 years; diagnosis of pituitary, hypothalamic, or neurodegenerative disorder (e.g., multiple sclerosis); metabolic disease (hyperglycemia, hyperlipemia, fatty liver, hyperinsulinemia, diabetes, and obesity); serum biochemical parameter abnormalities without definite metabolic disease (including CHOL, TG, HDL, LDL, and GLU); cerebral atrophy; and history of bariatric disease. Twenty subjects were excluded (age <16 [n = 2]; age >70 [n = 1]; metabolic disease [n = 5]; serum biochemical parameter abnormalities [n = 9]; CNS disease [n = 3]), yielding a total of 31 study participants. All subjects were classified into 2 groups: the hyperuricemia group (fasting serum UA >416 μM for men, >357 μM for women) and the normal group. The basal characteristics of the study population are provided in Supplementary Table S1. ROIs in the bilateral MBH and amygdala were defined by a neuroradiologist, who was blinded to all clinical information. Mean ROI signal intensity, standard deviation, and ROI area were measured using proprietary software on the PACS workstation (Centricity, GE Healthcare). Ratios were calculated by comparing the mean signal intensity in the MBH on each side with that in the ipsilateral amygdala ROI. Our study was approved by the Medical Ethics Committee (Human) of West China Hospital and Sichuan University. All subjects had been informed and approved participation in this study, and “informed consent” was obtained from all subjects. The methods were carried out in accordance with the approved guidelines.

### Statistical analysis

Data are presented as the mean ± SEM. Differences between two means were assessed by unpaired, two-tailed Student’s t test. Data involving more than two means were evaluated by one-way ANOVA followed by Tukey’s post-hoc tests (SigmaStat [SyStat] and GraphPad Prism [GraphPad Software, Inc.]). *P* values less than 0.05 were considered statistically significant.

## Additional Information

**How to cite this article**: Lu, W. *et al.* Uric Acid Produces an Inflammatory Response through Activation of NF-κB in the Hypothalamus: Implications for the Pathogenesis of Metabolic Disorders. *Sci. Rep.* **5**, 12144; doi: 10.1038/srep12144 (2015).

## Supplementary Material

Supplementary Information
