# Supplementary material for: Uric Acid Produces an Inflammatory Response through Activation of NF-κB in the Hypothalamus: Implications for the Pathogenesis of Metabolic Disorders
Source: Sci Rep. 2015 Jul 16;5:12144. doi: 10.1038/srep12144 (PMC4503982; doi:10.1038/srep12144)
Supplement: Supplementary Information [file srep12144-s1.pdf]

Supplementary Information

Uric Acid Produces an Inflammatory Response through Activation of NF-κB in the Hypothalamus: Implications for the Pathogenesis of Metabolic Disorders

Wenjie Lu[1,†], Youzhi Xu[2,†], Xiaoni Shao[1,†], Fabao Gao[3,†], Yan Li[1], Jing Hu[1], Zeping Zuo[1], Xue Shao[4], Liangxue Zhou[5], Yinglan Zhao[1,*] & Xiaobo Cen[4,*]

[1]State Key Laboratory of Biotherapy and Cancer Center, West China Hospital, Sichuan University, and Collaborative Innovation Center for Biotherapy, Chengdu 610041, China. [2]Department of Pathophysiology, School of Basic Medicine, Anhui Medical University, Hefei 230032, China. [3]Molecular Imaging Laboratory, Department of Radiology, West China Hospital, Sichuan University, Chengdu 610041, China. [4]National Chengdu Center for Safety Evaluation of Drugs, State Key Laboratory of Biotherapy and Cancer Center, West China Hospital, Sichuan University, and Collaborative Innovation Center for Biotherapy, Chengdu 610041, China. [5]Department of Neurosurgery, West China Hospital, Sichuan University, Chengdu 610041, China.

[†]These authors contributed equally to this work.

[*]Co-corresponding author, address correspondence to: Xiaobo Cen, National Chengdu Center for Safety Evaluation of Drugs, State Key Laboratory of Biotherapy and Cancer Center, West China Hospital, Sichuan University, and Collaborative Innovation Center for Biotherapy, #1 Keyuan Road 4, Gaopeng Street, High Technological Development Zone, Chengdu 610041, China. Phone: +86-28-85178767, Fax: +86-28-85173043, E-mail: xbcen@scu.edu.cn.

Supplementary information includes:

Supplementary Figure S1-S2;

Supplementary Table S1-S3;

Supplementary Methods;

Supplementary Discussion;

Supplementary References.

Supplementary Figure

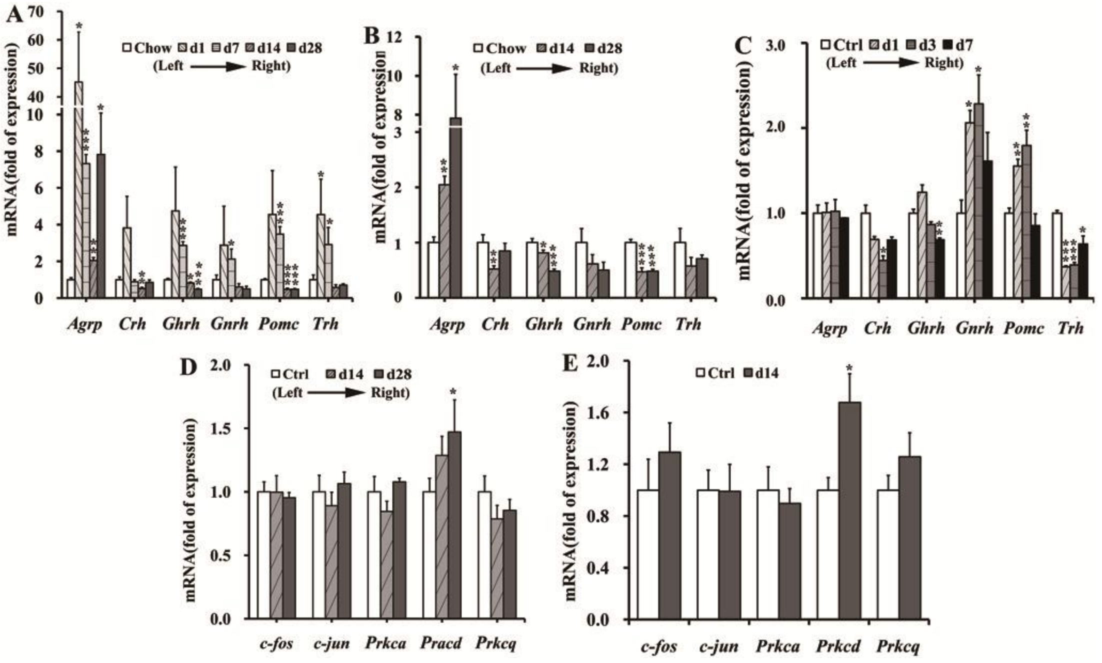

Supplementary Figure S1. The role of UA on the mRNA levels of relevant hypothalamic neurotransmitters and possible mediators. (A,B) Time course of changes in mRNA encoding relevant hypothalamic neurotransmitters, *Agrp*, *Pomc*, *Crh*, *Trh*, *Ghrh*, and *Gnrh*, in the hypothalamus of rats fed chow or a HUAD for 1, 7, 14, and 28 days (A) and in which multiple neurotransmitters decreased on day 14 and day 28 (B) (n = 6 rats per group). (C) Time course of changes of hypothalamic neurotransmitters in rats with icv injection of UA for 1, 3, and 7 days (n = 6 rats per group). (D,E) The mRNA levels of *c-fos*, *c-jun*, *Prkca*, *Prkcd*, and *Prkcq* were tested in the systemic hyperuricemic model (D) and icv-cannulated rats model (E) (n = 6 rats per group). All mRNA species were quantified relative to *Gapdh* housekeeping gene expression and are presented as fold changes relative to chow-fed controls or saline controls. *$P < 0.05$; **$P < 0.01$; ***$P < 0.001$ versus control.

A.

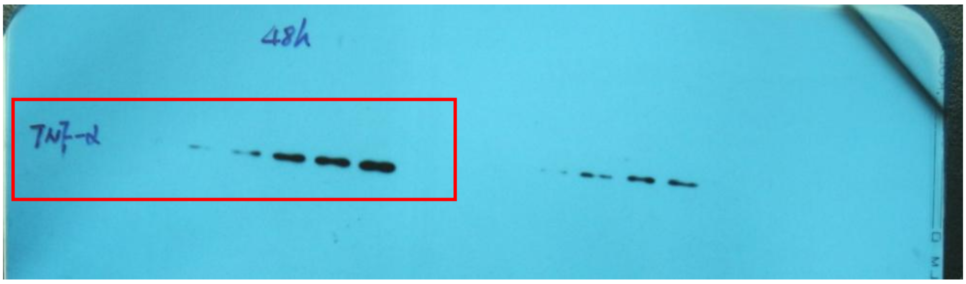

B.

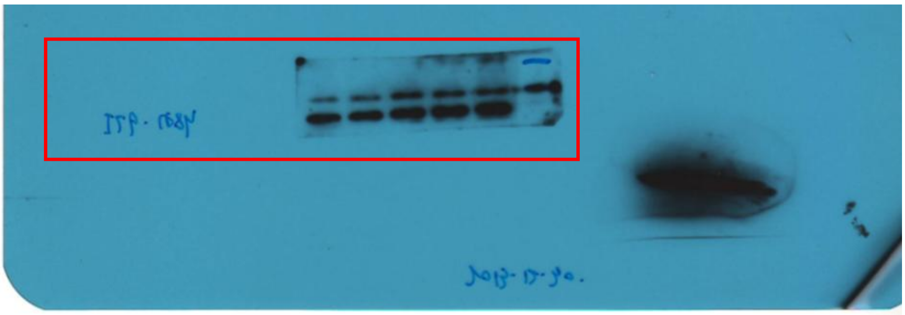

C.

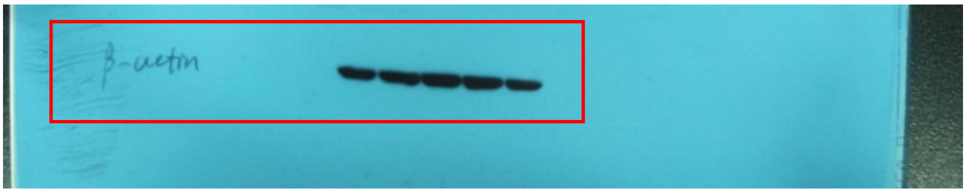

Supplementary Figure S2. Full unedited blots for TNF-α (A), IL-6 (B) and β-actin (C).

Supplementary Table

Supplementary Table S1. Basal characteristics of the study population comparing controls and hyperuricemia subjects.

	Control (n=16)	Hyperuricemia (n=15)	*P value*
Gender (F/M)	7/9	9/6	/
Age(years)	34.25±3.48	32.67±3.79	0.3802
Body weight (kg)	55.63±3.04	58.22±2.47	0.2955
UA(μmol/L) [a]	293.13±19.42	483.63±37.15	<0.001
TBIL(μmol/L)	10.58±1.14	12.66±1.87	0.3883
DBIL(μmol/L)	4.18±0.54	4.81±0.77	0.5453
IBIL(μmol/L)	6.40±0.67	7.85±1.14	0.3202
ALT(IU/L)	23.63±4.66	24.36±4.17	0.9188
AST(IU/L)	22.94±3.31	19.09±1.38	0.3624
TP(g/L)	67.63±1.07	69.26±4.59	0.4081
ALB(g/L)	42.43±0.65	45.04±3.00	0.0624
GLB(g/L)	25.19±0.93	24.14±1.79	0.4710
GLU(mmol/L)	4.89±0.15	4.85±0.32	0.8608
BUN(mmol/L)	5.74±0.56	4.90±0.39	0.2429
CREA(μmol/L)	68.13±3.90	78.77±6.06	0.1084
Cys-C(mg/L)	0.90±0.04	0.93±0.07	0.5769
TG(mmol/L)	1.24±0.24	1.27±0.17	0.9358
CHOL(mmol/L)	4.12±0.29	3.90±0.31	0.6044
HDL-C(mmol/L)	1.37±0.12	1.33±0.10	0.8354
LDL-C(mmol/L)	2.33±0.20	2.18±0.21	0.6076
ALP(IU/L)	68.50±5.54	91.64±16.00	0.2233
GGT(IU/L)	21.50±5.14	19.55±2.38	0.7719
CK(IU/L)	90.56±15.13	117.27±15.66	0.2877
LDH(IU/L)	156.25±9.33	168.91±13.94	0.4209
HBL(IU/L)	123.94±7.57	134.27±11.75	0.4437
Na(mmol/L)	141.78±0.56	142.35±9.13	0.4840
K(mmol/L)	3.92±0.08	3.89±0.26	0.8253
Cl(mmol/L)	106.31±0.83	105.14±6.82	0.3490
CO_2(mmol/L)	24.46±0.61	23.01±1.59	0.1739
β-HBA(mmol/L)	0.14±0.02	0.19±0.06	0.5113
Ca(mmol/L)	2.18±0.02	2.24±0.14	0.1452
Mg(mmol/L)	0.90±0.02	0.84±0.06	0.0457
PO_4(mmol/L)	1.20±0.04	1.21±0.12	0.8756

The values are represented as means ± SEM. UA indicates uric acid. A significant T-test ($P <$ 0.001, compared with control).

Supplementary Table S2. Primers used for gene expression analysis by qRT-PCR

Species	name	Sequence (Forward)	Sequence (Reverse)
	Nfkbia	TGCCTGGCCAGTGTAGCAGTCTT	CAAAGTCACCAAGTGCTCCACGAT
	Ikbkb	AGGGTGACTAAGTCGAGAC	ACAGCCAGGATATGGTACG
	Ikbke	ACCACTAACTACCTGTGGCAT	ACTGCGAATAGCTTCACGATG
	Il6	CAGAGGATACCACCCACAACAGA	CAGTGCATCATCGCTGTTCATACA
	Il1b	TACAAGGAGAGACAAGCAACGACA	GATCCACACTCTCCAGCTGCA
	Tnfa	GCTCCCTCTCATCAGTTCCA	CTCCTCTGCTTGGTGGTTTG
	Gfap	AACGACTATCGCGCACTG	CTCTCTGTTCGCGCATTG
	Gapdh	AACGACCCCTTCATTGAC	TCCACGACATACTCAGCAC
	Agrp	AGCAGACCGAGCAGAAGATGT	GGTATTGAAGAAGCGGCAGTAG
Rats	*Crh*	GAAACTGAAGAGAAAGGGGAAAG	TGAGCCCGCACTGTTGTT
	Ghrh	GGTGTTCTTTGTGCTCCTCAC	ATCTTTGTTCCTGGTTCCTCTC
	Gnrh	GCTGTTGTTCTGTTGACTGTGTG	CTCCTCCTTGCCCATCTCTT
	Pomc	CTATCGGGTGGAGCACTTC	GTTCTTGATGATGGCGTTCTT
	Trh	GTGAAGCAAAGCCCACAAGT	GGGAACAGGATAGGGAATACAG
	c-fos	CGTCTTCCTTTGTCTTCACCTA	CTGCCTTCTCTGACTGCTCAC
	c-jun	GCCTGCCTCTCTCAACTATGTA	TAGGACACCCAAACAAACAAAC
	Prkca	CCAAGAGGAGGGTGAATACTACA	GAAGGGCTGATGACTTTGTTTC
	Prkcd	CAGACAACAACGGGACCTATG	CTTTCCTTGCCCTTCAGTTCT
	Prkcq	CCACAGAGAAGGACCCACTAAC	TATTGGAGAACATCCCATCACA
	Nfkbia	TCGCTCTTGTTGAAATGTGG	CTCTCGGGTAGCATCTGGAG
	Il6	CGGAGAGGAGACTTCACAGAG	CATTTCCACGATTTCCCAGA
Mice	*Tnfa*	CCACCATCAAGGACTCAAATG	GAGACAGAGGCAACCTGACC
	Il1b	CTCACAAGCAGAGCACAAGC	TCCAGCCCATACTTTAGGAAGA

Supplementary Table S3. High-resolution MRI protocol for quantitative assessment of hypothalamic inflammation.

Method	Sequence type	TR/TE (ms)	FOV (mm)	Acquisition/ Reconstruction Resolutions	Acquisition Time
Triplot GE	FLASH	100/6	80 × 80	-	12 s
T2W	Multislice RARE	2600/33,<br>24 slices (slice thickness = 1 mm),<br>FA = 180 °	45 × 45	176 μm × 176 μm	1 min 24 s
T1W	Multislice, Multiecho	500/8,<br>24 slices (slice thickness = 1 mm),<br>FA = 180 °	45 × 45	234 μm × 234 μm	2 min 24 s
T2Map	Multislice, Multiecho	2200/11-110,<br>24 slices (slice thickness = 1 mm),<br>FA = 180 °	45 × 45	176 μm × 176 μm	7 min 2 s
DTI	EPI	4000/32,<br>30 diffusion directions,<br>b = 670 s/mm^2,<br>slice thickness = 1 mm,<br>FA = 90 °	45 × 45	351 μm × 351 μm	9 min 20 s

TR, recycle delay; TE, echo time; FOV, field of view; GE, gradient echo; FA, flip angle; RARE, rapid acquisition with refocused echoes; T1W, T1 weighted; T2W, T2 weighted; DTI, diffusion tensor imaging; EPI, echo planar imaging.

Supplementary Methods

Antibodies and chemicals. The primary antibodies for western blotting detection of IL-6, TNF-α, and β-actin as well as the HRP-conjugated secondary antibodies were purchased from Abcam (Cambridge, MA, USA). The primary antibodies for immunofluorescence analysis of Iba-1, GFAP, NF-κB (rabbit mAb), and NF-κB (goat mAb) were purchased from Wako Pure Chemicals (Osaka, Japan), Sigma-Aldrich (St. Louis, MO, USA), Cell Signaling Technology (Beverly, MA, USA), and Santa Cruz Biotechnology (Santa Cruz, CA, USA), respectively. Fluorescently labeled secondary antibodies were purchased from Zhong Shan Biotechnology (Beijing, China), Abcam (Cambridge, MA, USA), and Sigma-Aldrich (St. Louis, MO, USA). Hoechst 33258 was from Sigma-Aldrich (St. Louis, MO, USA). A commercial kit for the extraction of total mRNA from the hypothalamus was purchased from Axygen Scientific (CA, USA). The reverse transcription kits and fluorescence quantification kits for qRT-PCR were purchased from Bio-Rad (Hercules, CA, USA). The primers for qRT-PCR were designed and synthesized by Sangon Biotech (Shanghai, China). The reagents for serum biochemical analysis were purchased from Maker Science & Technology (Chengdu, China). UA, oxonic acid and allantoin were purchased from Chemlin Chemical Industry (Nanjing, China) and BAY11-7085 was purchased from TCI Industrial Development Company (Shanghai, China). All of the chemicals employed in this study were of analytical grade.

qRT-PCR. RNA was extracted from the hypothalamus using a commercial kit

according to the manufacturer's instructions. RNA was quantified by spectrophotometry at 260 nm and reverse transcribed with a reverse transcription kit (Bio-Rad). Levels of mRNA for *Nfkbia*, *Ikbkb, Ikbke, Il6, Il1b, Tnfa*, *Ccl2, Crp, Gfap, Agrp*, *Pomc*, *Crh*, *Trh*, *Ghrh*, *Gnrh*, *c-fos*, *c-jun*, *Prkca*, *Prkcd*, *Prkdq*, and *Gapdh* (internal control) were measured by qRT-PCR (Bio-Rad). The expression level of each gene was normalized to a housekeeping gene (*Gapdh*) and expressed as a percentage of chow-fed control or saline control. The primer sequences are provided in **Supplementary Table S2**.

Immunostaining. After fixation with 4% paraformaldehyde, paraffin-embedded coronal sections through the hypothalamus were processed for immunoreactivity using standard immunofluorescence procedures. Sections were incubated overnight at 4 ℃ with mouse anti-GFAP (1:500; Sigma-Aldrich) or rabbit anti-Iba-1 (1:500; Wako Pure Chemicals). Immunofluorescence was performed with a combination of Alexa Fluor 488- or Alexa Fluor 594-labeled anti-rabbit or anti-mouse secondary antibodies and Hoechst 33258, which was used to identify cell nuclei. Images were captured on a microscope equipped with a color digital camera (Nikon, Japan). For Iba-1 and GFAP immunostaining in which discrete cells could be identified, cells were manually counted on 3 slides per rat, and replicate values from each rat were individually averaged before determining group means (6 rats per group).

UA determination in CSF and hypothalamic tissue. After the rats were

anesthetized and cerebrospinal fluid collected from the foramen magnum, hypothalamic tissue was collected. UA concentrations in the CSF and hypothalamic tissue were quantified by liquid chromatography tandem mass spectrometry (LC-MS/MS)[1]. The LC-MS/MS was composed of a Shimadzu high-performance liquid chromatography system consisting of two LC-20ADXR pumps (Shimadzu, Kyoto, Japan) and an AB Sciex 3200 QTRAP mass spectrometer (Framingham, MA, USA). CSF samples were analyzed immediately after centrifugation. For hypothalamic tissue samples, 4-5 samples were mixed as a pool, water was added (v/m 1:4), and the sample was homogenized.

Liquid chromatography analyses were performed using a Phenomenex Synergi Hydro-RP 4 μm column (2.0 mm × 50 mm). The mobile phases used included an acetonitrile and water phase (10 mM ammonium acetate at pH 4.5), with a 5:95 volume ratio in the isocratic elution mode. The flow rate was 0.3 ml/min, and the injection volume was 5 μl.

UA was analyzed in negative ESI mode with collision energy of -20 V. The turboheater temperature was set at 600 ℃. The nebulizing gas, turboheater gas, and curtain gas flow rates were 45, 60 and 10, respectively. Ion spray voltage was -4 kV. The ions monitored for MRM were as follows: the parent ion of UA was m/z 166.9 and the monitored MRM ion was m/z 123.9. Using this LC-MS/MS method, the concentration of UA is linear from 10 to 8000 ng/ml.

Hypothalamic primary cultures. Primary hypothalamic neural cells were prepared

from fetal rat brains obtained on embryonic day 18[2]. Briefly, hypothalami were dissected and stored on ice in DMEM/F12 (Hyclone) supplemented with 10% horse serum (GIBCO). Tissues were transferred into 0.125% trypsin (GIBCO), and incubated for 15-30 min in 37 ℃. The trypsin was removed, and DMEM/F12 was added to terminate digestion. Tissues were then gently pipetted 10-15 times, and uniform cellular dissociation was achieved. Cells were seeded into 6-well culture plates coated with poly-L-lysine (Sigma-Aldrich). After 4-6 h, the medium was changed to adult neuronal growth medium consisting of neurobasal medium (Invitrogen), B-27 supplement (Invitrogen), and 0.5 mM L-glutamine (GIBCO).

Immunofluorescence for primary cultured cells. Primary hypothalamic neurons and glial cells were stained as described. In brief, the cells were fixed with 4% paraformaldehyde for 5 min, followed by penetration with Triton-X 100 (Invitrogen). After blocking with 5% BSA (Sigma), the cells were incubated with primary antibodies to NeuN (1:100, Millipore), GFAP (1:500, Sigma-Aldrich), Iba-1 (1:200, Wako Pure Chemicals), and p65/RelA (1:100, Cell Signaling Technology) at 4 ℃ overnight. After washes, the cells were incubated with the appropriate secondary antibody at 37 ℃ for 2 h. Images were captured on a microscope equipped with a color digital camera (Nikon, Japan).

Cell cultures. SH-SY5Y human neuroblastoma cells were purchased from the American Type Culture Collection (Manassas, VA, USA). Cells were cultured in

DMEM/F12 (v/v 1:1) supplemented with 10% FBS (GIBCO) and 2 mM glutamine at 37 ℃ in a 5% CO_2 atmosphere and were passaged 2-3 times before use.

Western blotting analysis. Western blotting analysis was performed according to standard methods. Briefly, lysate protein concentrations were equalized, resolved on sodium dodecyl sulfate polyacrylamide gel electrophoresis gels, and transferred to nitrocellulose membranes. The gels were run under the same experimental conditions. Proteins were detected with the appropriate primary and secondary antibodies, and protein bands were visualized using an enhanced chemiluminescence kit (Merck Millipore, Billerica, MA, USA). Data typical of 3 independent experiments are shown.

Brain MRI in rats. Wistar rats (6 weeks old) were fed standard laboratory chow or a HUAD for 3 months. High-resolution MRI acquisitions were performed on a 7T Avance 600 MHz/89 mm wide-bore vertical MR spectrometer (Bruker BioSpin, Billerica, MA) using a 25 mm inner diameter ^{1}H birdcage coil. The 7T MRI system was equipped with actively shielded gradient coils (maximum gradient strength of 100 G/cm) and a Paravision (version 5.1) console interface.

All rats underwent isoflurane anesthesia in an induction chamber. The rats were placed on a bite bar, and their heads were placed into a radiofrequency coil and secured to a cradle created specifically for the MRI system. The coil was then inserted vertically into a scanner heated to maintain thermoneutrality (32 ℃). The coil was

equipped with an adjustable anesthetic flow and vacuum system to maintain sedation throughout the experiment. Total scan time was 1-1.5 h, during which anesthesia was titrated to ensure appropriate sedation. Following the imaging paradigm (described in **Supplementary Tab. S3**), mice were removed from the coil and allowed to recover in their home cage[3,4].

Biochemical measurements. During the experimental period, the rats were fasted overnight in advance of sampling, and blood samples were obtained by cardiac puncture. Serum biochemical analysis was performed with a Hitachi 7020 Automatic Analyzer (Hitachi, Tokyo, Japan) and included ALT, AST, ALP, ALB, TBIL, TP, UREA, CREA, GLU, CK, TG, CHOL, LDL, HDL, and UA.

Serum insulin quantification. Serum insulin was quantified using a commercial ELISA kit from Merck Millipore (Billerica, MA, USA) according to the recommendations of the manufacturer.

Oral glucose tolerance test (OGTT). An OGTT was performed after the rats were fed a chow diet or the HUAD for 1, 2, and 3 months. In the icv-cannulated rats, the OGTT was performed on the 7th and 14th day of the experimental period, and the detection was carried out 24 h after administration of the last dose of UA. In the mice, the OGTT was carried out 8 h after the last dose of UA and BAY11-7085. A steady-state blood sample was taken from the tip of the tail after 8 h of food

withdrawal, and serum glucose was measured using a glucose meter (Omron, Beijing, China). Subsequently, each animal received a glucose solution load (1 g/kg for rats, 2 g/kg for mice, orally), and additional blood samples were measured at 15, 30, 60, 90, and 120 min after the injection. An AUC was calculated from the values of each animal.

Supplementary Discussion

Here we report that rats with systemic hyperuricemia exhibited hypothalamic inflammation, which is a manifestation of neuronal injury that in turn triggers gliosis that involves both microglial and astroglial cell populations. We found that UA in serum can pass through the BBB and induce potent inflammation as well as gliosis in the hypothalamus. Hypothalamic NF-κB activation mainly occurred in neurons, and NF-κB inhibition was able to protect against UA-induced hypothalamic inflammation in the mice. Importantly, MRI showed that hypothalamic gliosis was closely associated with hyperuricemia in rodents and humans, raising the possibility of a common mechanism of UA-induced hypothalamic inflammation across species. Finally, rats fed a HUAD exhibited dyslipidemia and glucose intolerance, which is likely mediated by hypothalamic inflammation and neuroendocrine alterations.

In addition to its role as an inflammatory regulator, NF-κB signaling also controls cell survival, apoptosis and synaptic plasticity[5]. More specifically, during nervous system development, NF-κB is activated in growing neurons by neurotrophic factors and induces the expression of genes involved in cell differentiation and survival[6]. NF-κB can also induce the differentiation of neuronal stem cells into astrocytes depending on the signaling cascade[7] and can prevent the death of neurons by inducing the production of antiapoptotic proteins in acute neurodegenerative conditions[8,9]. The involvement of NF-κB signaling in dendritogenesis, dendritic arborization and axonal growth[10] has been well documented, as has its mediation of myelination in Schwann cells[11].

A metabolic disorder phenotype can be induced by hypothalamic dysfunction; however, how hypothalamic dysfunction controls peripheral metabolic organs is still uncertain. Indeed, we revealed a direct link between UA and neuropeptides as well as neuroendocrine factors, indicating that UA suppresses the expression of *Pomc*, *Crh*, *Trh*, *Ghrh*, and *Gnrh*. The importance of *Pomc*, *Trh*, and *Crh* in the regulation of thermogenesis and glucose consumption in BAT and skeletal muscle has been well established[12]. Hypothalamic immunity mediated by IKKβ/NF-κB can inhibit neuroendocrine factors, such as gonadotropin-releasing hormone[12]. Based on these findings, we propose that UA causes hypothalamic inflammation and NF-κB activation, at least in part, in POMC and AgRP neurons, which directly influences feeding and glucose homeostasis through the autonomic nervous system. On the other hand, the neurons that release neurotransmitter may have direct synaptic connections with the neurons that release hypothalamic regulatory peptides[13]. Although the present study did not explore the mechanisms that effectively deliver the neural signal to the affected peripheral organs, it is tempting to propose that both the autonomic nervous system and hormones could be involved.

Supplementary References

1 Dai, X., Fang, X., Zhang, C., Xu, R. & Xu, B. Determination of serum uric acid using high-performance liquid chromatography (HPLC)/isotope dilution mass spectrometry (ID-MS) as a candidate reference method. *J Chromatogr B Analyt Technol Biomed Life Sci* **857**, 287-295 (2007).

2 Kim, M. S. *et al.* Role of hypothalamic Foxo1 in the regulation of food intake and energy homeostasis. *Nat Neurosci* **9**, 901-906 (2006).

3 Thaler, J. P. *et al.* Obesity is associated with hypothalamic injury in rodents and humans. *J Clin Invest* **122**, 153-162 (2012).

4 Lee, D. *et al.* Longer T(2) relaxation time is a marker of hypothalamic gliosis in mice with diet-induced obesity. *Am J Physiol Endocrinol Metab* **304**, E1245-1250 (2013).

5 Mattson, M. P. & Meffert, M. K. Roles for NF-kappaB in nerve cell survival, plasticity, and disease. *Cell Death Differ* **13**, 852-860 (2006).

6 Sarnico, I. *et al.* NF-kappaB dimers in the regulation of neuronal survival. *Int Rev Neurobiol* **85**, 351-362 (2009).

7 Kaltschmidt, B. & Kaltschmidt, C. NF-kappaB in the nervous system. *Cold Spring Harb Perspect Biol* **1**, a001271 (2009).

8 Charalampopoulos, I. *et al.* Dehydroepiandrosterone and allopregnanolone protect sympathoadrenal medulla cells against apoptosis via antiapoptotic Bcl-2 proteins. *P Natl Acad Sci USA* **101**, 8209-8214 (2004).

9 Pizzi, M. *et al.* NF-kappaB factor c-Rel mediates neuroprotection elicited by mGlu5 receptor agonists against amyloid beta-peptide toxicity. *Cell Death Differ* **12**, 761-772 (2005).

10 Gutierrez, H. & Davies, A. M. Regulation of neural process growth, elaboration and structural plasticity by NF-kappaB. *Trends Neurosci* **34**, 316-325 (2011).

11 Memet, S. NF-kappaB functions in the nervous system: from development to disease. *Biochem Pharmacol* **72**, 1180-1195 (2006).

12 Cypess, A. M. *et al.* Identification and importance of brown adipose tissue in adult humans. *New Engl J Med* **360**, 1509-1517 (2009).

13 Verberne, A. J., Sabetghadam, A. & Korim, W. S. Neural pathways that control the glucose counterregulatory response. *Front Neurosci* **8**, 38 (2014).
